# Predominance of OXA-48 Carbapenemase-Producing Enterobacterales in a Moroccan Hospital

**DOI:** 10.1155/2023/8581883

**Published:** 2023-05-19

**Authors:** El Mehdi Belouad, Elmostafa Benaissa, Nadia El Mrimar, Fatna Bssaibis, Adil Maleb, Mostafa Elouennass

**Affiliations:** ^1^Department of Clinical Bacteriology, Mohammed V Military Teaching Hospital, Rabat, Morocco; ^2^Research Team of Epidemiology and Bacterial Resistance, Faculty of Medicine and Pharmacy, Mohammed V University in Rabat, Rabat, Morocco

## Abstract

**Objective:**

The emergence of carbapenemase-producing Enterobacterales (CPE) is a major concern that is increasingly reported worldwide. Our study aimed at investigating the resistance of CPE isolates in a Moroccan teaching hospital using phenotypic and genotypic methods.

**Methods:**

Enterobacterales strains from March to June 2018 were collected from different clinical samples. The Enterobacterales isolates resistant to third-generation cephalosporins (3GC) and/or carbapenems were subjected to the Carba NP test and an immunochromatographic test for phenotypic detection. Detection of extended-spectrum *β*-lactamases (ESBL) was also performed following standards. Molecular screening of carbapenemases genes (OXA-48, NDM, blaKPC, blaIMP, blaVIM, and blaOXA-24, blaOXA-23, OXA-51, OXA-58) using conventional multiplex PCR assays was also performed on 143 isolates.

**Results:**

Enterobacterales represented 52.7% with a proportion of 21.8% of bacteria resistant to 3GC and/or carbapenems. Within 143 isolates MDR to 3GC, *K. pneumoniae*, *E. coli*, and *E. cloacae* represent 53.1%, 40.6%, and 6.3%, respectively. These strains were isolated mainly from urinary samples (74.8%) in patients admitted to emergency and surgical units. 81.1% of strains are producing ESBL and 29% are carbapenemase producers as confirmed by the Carba NP test, immunochromatographic test, and molecular testing. OXA-48 carriers represent 83.3% of these strains, followed by NDM with 16.7%. blaKPC, blaIMP, blaVIM, and blaOXA-24, blaOXA-23, OXA-51, OXA-58 were not detected in any of these bacteria.

**Conclusions:**

A high rate of CPE carrying OXA-48 among Enterobacterales resistant to 3GC and/or carbapenems isolates was found. Strict observance of hospital hygiene measures and more rational use of antibiotics are mandatory. Implantation of carbapenemases detection should be encouraged in our hospital settings to estimate the true burden of the CPE.

## 1. Introduction

Enterobacterales belong to a large order of different types of Gram-negative bacteria that commonly cause infections in humans typically found under healthcare settings. Enterobacterales are highly susceptible to carbapenems, a class of beta-lactam antibiotics, which include penicillins, cephalosporins, and monobactams [1]. Interestingly, carbapenems demonstrate a broader spectrum of activity than other beta-lactams and have always been considered as the last line of defense in the treatment of multidrug-resistant Gram-negative (MDR) bacterial infections [2, 3]. However, resistance to carbapenems over the last 10 years has become a critical global challenge in the health sector. Consequently, there are few therapeutic interventions for treating severe infections caused by carbapenem-resistant bacteria, such as *Klebsiella pneumoniae* and *Escherichia coli* [4, 5].

Furthermore, this acquired carbapenem resistance is attributed to either of the following two mechanisms: (a) the structural mutation combined with *β*-lactamase production and (b) the production of carbapenemases which hydrolyze carbapenems [6]. Generally, carbapenemases are classified into three among four classes according to the Ambler *β*-lactamase classification system. Carbapenemases belonging to class A are termed *β*-lactamases (*K. pneumoniae* carbapenemase (KPC)), which are inhibited by clavulanic or boronic acids, while class B are metallo-*β*-lactamases (MBLs) (New Delhi metallo-*β*-lactamase (NDM), imipenemase (IMP), and verona integron-encoded metallo-*β*-lactamase ((VIM)) and are inhibited by chelating agents, such as EDTA and dipicolinic acid, and lastly class D, which are known as *β*-lactamases (oxacillinases), including OXA-48-like enzymes. Notably, these enzymes are not inhibited by classical inhibitors [7, 8]. Individuals with compromised immune system are highly susceptible to infections caused by CPE, including those with chronic comorbid conditions or poor functional status. In addition, the abuse of antibiotics is considered the main initiator in the development of CPE [1, 9].

Some of the infections caused by CPE are reported in the last review of van Duin and Doi [1]. Briefly, the KPC family is the most predominant among all carbapenemases in CPE and is common in USA, as well as in Central and Latin America. In Europe, the highest incidence of KPC was found in Italy and Greece, and a pandemic was also reported in China [7].

For MBL-producing CPE, they are mainly associated with the Indian subcontinent (India, Pakistan, and Bangladesh), of which *bla*NDM-1 is a concern. VIM is the predominant MBL in Europe, especially Poland, Romania, and Denmark. The distribution of carbapenemases in the OXA-like class is mostly prevalent in the Mediterranean countries in Turkey and surrounding countries, which are considered the epicenters of OXA-48-like-producing CPE. Furthermore, these OXA-48-like enzymes are distributed in the Middle East, Africa, and South America [10, 11].

In Morocco, data on CPE are limited as related patients are not systematically investigated in healthcare settings. This is partly attributed to the absence of a national surveillance program, and most clinical microbiology laboratories do not perform molecular testing, which is the reference standard for the identification and differentiation of carbapenemases. Consequently, there are still insufficient data on CPE in patients and co-carriers. In addition, in the few existing reports, there is no information on the different classes of CPE. Consequently, the existing data are likely inconclusive.

In this study, we performed a retrospective study to detect carbapenemase producers in clinical specimens via conventional PCR technique with different multiplex of primers.

## 2. Materials and Methods

### 2.1. Study Design

This study was carried out at the Bacteriology Department of the Mohammed V Military Teaching Hospital in Rabat, Morocco. This hospital serves the healthcare needs of approximately 1,800,000 patients per year. Our laboratory performs the bacteriological exams of almost 60,000 samples per year.

Between March and June 2018, clinical isolates (*n* = 2875) were identified in the current study. They were obtained from various clinical samples (urine, pus, blood, respiratory samples, and body fluid aspirates) collected from 915 inpatients and 1079 outpatients.

### 2.2. Bacterial Strains and Microbiology Methods

The various clinical specimens were cultured at 37°C aerobically for 18 to 24 hours on blood agar plates except for urine specimens that were cultured on CLED agar plate. Gene identification was performed by the standard bacteriological and biochemical methods using API-20E (bioMérieux SA, Marcy-l′Étoile/France) ready-to-use strips.

### 2.3. Antibiotic Susceptibility Testing

The assay was performed by the Mueller-Hinton agar diffusion method using Oxoid® antibiotic discs and interpreted according to EUCAST/CA-SFM 2019 recommendations [12]. Thirteen antibiotics (bioMérieux, Marcy L'Étoile, France) were tested, namely, ampicillin (AMP), amoxicillin-clavulanic acid (AMC), piperacillin tazobactam (TZP), mecillinam (MEC), cefixime (CFM), ceftriaxone (CRO), cefoxitin (FOX), ertapenem (ETP), imipenem (IPM), gentamicin (GEN), amikacin (AN), norfloxacin (NOR), trimethoprim/sulfamethoxazole (STX), and fosfomycin (FOS). The reading of the antibiotic susceptibility testing was performed using the Adagio™ Bio-Rad® system. Quality control of the antibiotic susceptibility testing was performed by Thermo Scientific™ Culti-Loops™*Escherichia coli* ATCC™ 25922™.

### 2.4. Defining Inclusion Criteria of a Given MDR Isolate

A MDR case was defined as the first clinical culture resistant to 3GC and/or carbapenems of the Enterobacterales family in a specified individual. The MDR positive samples for the same strain presenting similar antibiotic susceptibility profiles for the same patient were excluded. In addition, only patients with medical subscriptions for CPE screening were included (127 patients and 143 isolates).

### 2.5. Detection of Extended-Spectrum *β*-Lactamases (ESBL)

The detection of extended-spectrum *β*-lactamases (ESBL) was performed by a phenotypic method based on the detection of synergy between the amoxicillin-clavulanic acid disc and three third-generation cephalosporin discs [13].

### 2.6. Carba NP Test ®

The phenotypic detection of carbapenemases was performed using the Carba NP test® [14]. This test uses chromogenic medium based on phenol red as a pH indicator, and the product of imipenem hydrolysis by carbapenemases causes the acidification of an indicator solution of phenol red that changes its color due to pH modification. Two solutions (A and B) were used, the A solution was prepared by adding phenol red (0.05%) and ZnSO_4_.7H_2_O (0.1 mmol/L) to ultrapure water, and the pH was adjusted to 7.8 ± 0.1. The B solution was freshly prepared by adding 12 mg/ml imipenem-cilastatin injectable to A solution. A calibrated loop (10 *μ*l) of bacterial colony cultured for 18 to 24 h on trypticase soy agar was resuspended in 200 *μ*l of Tris-HCl 20 mmol/L. Bacterial lysate (100 *μ*l) was added to two Eppendorf tubes labeled “A” and “B” containing 100 *μ*l of solution A and B, respectively, and incubated at 37°C and readings were taken within 2 hours. The test was considered positive when tube “A” was red and tube “B” was orange/yellow, and the test was considered negative when both tubes remained red. Quality control was achieved using *E. coli* 25922 as a negative control. Positive control consists of a local NDM-producing *Klebsiella pneumoniae* isolate which was confirmed by Cepheid Xpert® Carba-R test (Cepheid, Sunnyvale, USA).

### 2.7. Immunochromatographic Tests

Immunochromatography test RESIST-5 O.O.K.N.V (Coris BioConcept, Belgium) was performed as recommended by the manufacturer. It is a multiplex assay which detects five types of carbapenemases (OXA-163, OXA-48, NDM, KPC, and VIM). Briefly, from a fresh culture, 1-2 colonies are taken with a loop and then suspended in a semirigid tube containing 12 drops of LY-A buffer supplied with the kit. After homogenization, three drops were put in each one of two cassettes.

### 2.8. Molecular Characterization

#### 2.8.1. Whole Bacteria Preparation

Bacteria were grown overnight in agar plates. 1-2 colonies were diluted in ultrapure water (200 *μ*l) and heat-killed at 100°C for 10 minutes and then centrifuged at 14000 rpm for 5 min and the supernatant was collected (chromosomal/plasmid DNA). Genomic DNA (gDNA) yield was quantified by OD reading at 260 nm using a Plus spectrophotometer (Molecular Devices). gDNA was aliquoted into multiple tubes at 50 *μ*l volume and stored at −20°C and used in a short time frame (maximum of four weeks).

#### 2.8.2. Detection of blaKPC, blaOXA-23-Like, OXA-24-Like, blaOXA-48-Like, OXA-51-Like, OXA-58-Like, blaVIM Type, VIM1, VIM2, NDM-1, NDM, and RepA Genes

PCR analysis was performed using the primers listed in Table 1. Five multiplex PCRs were performed with different primer mix and were defined as multiplex 1–5 for primer mix of *bla*_KPC_, KPC, *bla*_OXA-48-like_, RepA genes; *bla*_VIM_ type, VIM2, NDM-1 genes; *bla*_NDM-1_, *bla*_IMP_ variant genes (except, IMP-3, IMP-16, IMP-27, IMP-31, IMP-34, and IMP-35) with *bla*_OXA-23-like_; OXA-58-like, VIM1, NDM genes; and OXA-23-like, OXA-24-like, and OXA-51-like genes, respectively.

PCR amplification for carbapenemase-encoding genes was performed in a final volume of 25 *μ*l. Reaction mixtures contained 5 *μ*l of 5 × MyTaq Reaction Buffer (Bioline, UK), 2.5 U of MyTaq™ DNA polymerase (Bioline, UK), and forward and reverse primers are used at a final concentration of 0.2 *μ*M each and 3 *μ*l of extracted DNA (10–100 ng).

The amplification procedure was as follows: predenaturation at 95°C for 10 min; 40 cycles of denaturation at 95°C for 15 s, various annealing conditions (Table 1) for 15 s, and extension at 72°C for 10 s. PCR was used on a thermal cycler (MyCycler™, Bio-Rad, Germany). Electrophoresis on 1% agarose gel was used to analyze the amplicons and visualize them under UV light.

### 2.9. Statistical Analysis

Statistical analysis was performed with SPSS version 25 (SPSS Inc., Chicago, IL, USA).

## 3. Results

**Figure 1:**
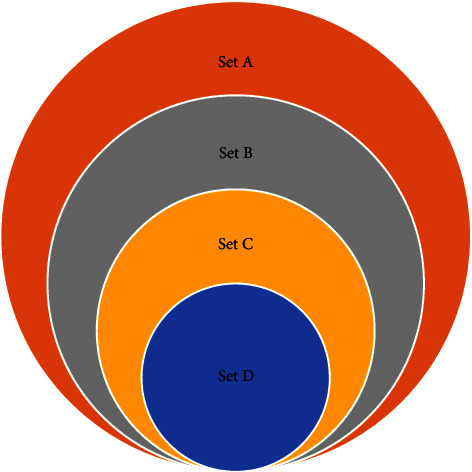
A Venn diagram represents four sets of isolates, illustrated here as colored circles. The orange circle, set A, represents all types of bacteria collected during the study period. The blue circle, set B, depicts the proportion of the Enterobacterales. The yellow circle, set C, illustrates the proportion of Enterobacterales that are resistant to third-generation carbapenems (3GC) and/or carbapenems and the blue set represents the portion of set C which benefited phenotypic and molecular screening of CPE.

Figure 1 illustrates the total clinical isolates (set A) collected during the study period (*n* = 2875 isolates) of which Enterobacterales (set B) represented 52.7% (*n* = 1516) with a proportion of Enterobacterales resistant to 3GC and/or carbapenems equals to 21.8% (*n* = 330) (set C). Almost half of these resistant strains (set D) have been subjected to phenotypic and molecular screening of CPE (*n* = 143).

**Figure 2:**
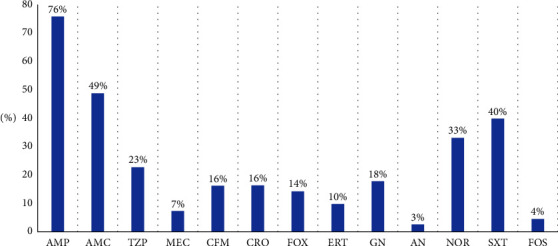
Antibiotic resistance profile of Enterobacterales (*n* = 1516) AMP: ampicillin; AMC: amoxicillin + clavulanic acid; TZP: piperacillin + tazobactam; MEC: mecillinam; CFM: cefixime; CRO: ceftriaxone; FOX: cefoxitin; ERT: ertapenem; GN: gentamicin; AN: amikacin; NOR: norfloxacin; STX: cotrimoxazole; FOS: fosfomycin.

Enterobacterales in set B included 61.2% (*n* = 928) of *E. coli*, 21.3% (*n* = 323) of *K. pneumoniae*, *Enterobacter cloacae* representing only 7% (*n* = 106), and the other species of Enterobacterales representing 10.4% (*n* = 159).  Figure 2 depicts the frequency of antibiotic susceptibility profiles of bacteria in set B. 76% of Enterobacterales were resistant to ampicillin, 49% to amoxicillin-clavulanic acid, and 40% to trimethoprim/sulfamethoxazole, and interestingly, only 3%, 4%, and 7% were not susceptible to amikacin, fosfomycin, and mecillinam, respectively. About 10% and 16% were resistant to ertapenem and ceftriaxone, respectively. The frequency of isolation of ESBL-producing Enterobacterales isolated during the study period was 16.3%.

The distribution of multiresistance phenotype within species in set C showed a predominance of *K. pneumoniae* (*n* = 135; 40.9%), followed by *E. coli* (*n* = 116; 35.2%), *E. cloacae* (*n* = 53; 16%), and other species of Enterobacterales (*n* = 26; 7.9%).

**Figure 3:**
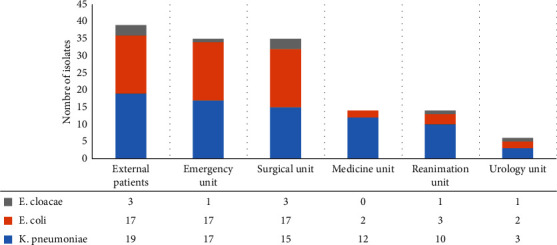
Distribution of multidrug resistant Enterobacterales in set D according to different hospital services.

The isolates in set D were mainly *K. pneumoniae* (*n* = 76) and *E. coli* (*n* = 58) representing 53.1% and 40.6%, respectively, and the remaining isolates were *E. cloacae* (*n* = 9). These strains were isolated from 127 patients with a mean age of 55.4 ± 20.3 years. The majority composed of inpatients (*n* = 109; 76%) and were mainly hospitalized in emergency and surgical units (Figure 3).

Most strains were isolated from urine (*n* = 107; 74.8%), pus (*n* = 24; 16.8%) blood (*n* = 8; 5.6%), respiratory tract (*n* = 3; 2.1%), and body fluid aspirates (*n* = 1; 0.7%).

**Figure 4:**
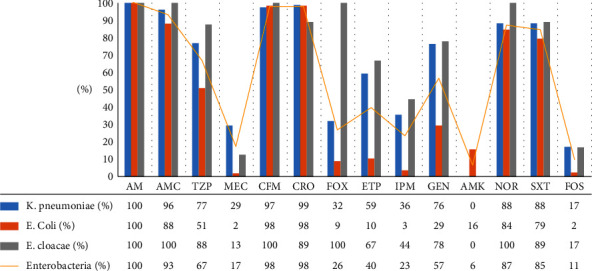
Resistance of Enterobacterales (set D) to 3GC and/or carbapenems (*n* = 143). AMP: ampicillin; AMC: amoxicillin + clavulanic acid; TZP: piperacillin + tazobactam; MEC: mecillinam; CFM: cefixime; CRO: ceftriaxone; FOX: cefoxitin; ERT: ertapenem; IPM: imipenem; GN: gentamicin; AN: amikacin; NOR: norfloxacin; STX: trimethoprim/sulfamethoxazole; FOS: fosfomycin.

Regarding the resistance rate of antibiotics in set D, all isolates were subjected to susceptibility testing for more characterization. They were resistant in 98% (*n* = 140) of cases to ceftriaxone, in 87% (*n* = 125) to norfloxacin, in 85% (*n* = 121) to trimethoprim/sulfamethoxazole, in 67% (*n* = 92) to piperacillin-tazobactam, in 57% (*n* = 82) to gentamicin, in 40% (*n* = 57) to ertapenem, in 23% (*n* = 31) to imipenem, and in 6% (*n* = 9) to amikacin (Figure 4).

Out of MDR Enterobacterales collected in set D, 81.1% of strains (*n* = 116) were producing ESBL where *E. coli*, *K. pneumoniae*, and *E. cloacae* strains represented 98% (*n* = 57), 72% (*n* = 55), and 44% (*n* = 4), respectively.

Concerning carbapenemase phenotype production, only 31.5% (*n* = 45) were detected by the Carba NP test. 47.4% (*n* = 36) of *K. pneumoniae* were phenotypically producing carbapenemases and all *E. cloacae* strains (*n* = 9; 100%) with no *E. coli* isolates.

Molecular analyses showed that only 29.4% of strains in set D (*n* = 42) harbored carbapenemase-mediating gene. Almost 83.3% of these strains carried OXA-48, followed by NDM with a percentage of 16.7%. blaKPC, blaIMP, blaVIM, and blaOXA-24, blaOXA-23, OXA-51, OXA-58 were not detected in the tested strains.

*K. pneumoniae* has the higher rate of carbapenemase-producing strains with 85.7% (*n* = 36; (29 OXA-48, 7 NDM)), followed by *E. cloacae* with 11.1% (*n* = 5; 5 OXA-48) and *E. coli* with a proportion of 2% (1 OXA-48) (Table 2).

A positive correlation of 95% was found between PCR and the flow lateral testing (Table 3).

The RepA replicase gene was detected in 32 (22.3%) isolates of which 30 (93.7%) were also positive for OXA-48. Five isolates had OXA-48 without expressing the RepA gene.

## 4. Discussion

The emergence of carbapenem resistance among Enterobacterales is an urgent threat to public health due to its current rapid global spread and the high morbidity and mortality rates [22]. During the study period, a high rate of Enterobacterales isolates was observed, consisting mainly of *E. coli*, followed by *K. pneumoniae* and *E. cloacae*. Moreover, these germs have been identified in comparable rates by Kone et al. at the Ibn Sina Hospital of Rabat [23]. Furthermore, these bacteria isolated during the study periode showed a resistance rate of 16% to 3GC and of 10% to ertapenem. Nevertheless, the resistance to 3GC was higher in Nigeria [24] (16% vs. 44.2%), but similar to ertapenem (10 vs. 6.5%). By comparing our data to those obtained in the United States [25], one can infer that our data are very high (16% vs. 1.6% for 3GC). Unlike the previous studies, our results for the carbapenem-resistant Enterobacterales concur with those reported by Loqman et al. (8.2%) in Morocco and Jalalvand et al. (13.6%) in Iran [26, 27].

According to the CASFM/EUCAST recommendations [12], the phenotypic algorithm for screening CPE strains required the strain to be carbapenem nonsusceptible. However, Karlowsky et al. [28] reported the presence of various carbapenemases, such as blaVIM, blaOXA-48-like, blaKPC, blaIMP, and blaGES in ertapenem-susceptible and ESBL-positive isolates. Consequently, we considered the resistance to 3GC as an inclusion criterion in our study and also to explore the production of carbapenemases among MDR phenotype. Our results showed that Enterobacterales were resistant to 3GC and/or carbapenems in 21.8% of cases, and this rate is high compared to the results obtained by Gupta et al. [25], which showed that the rate of Enterobacterales with an MDR phenotype was 6.9% in the United States. Among the MDR phenotype, *K. pneumoniae* is the most commonly isolated species. This result is consistent with those reported in earlier studies [26, 27, 29–31] and contrary to the findings of Colot et al. [32], according to which the most frequent species are *E. cloacae*.

Importantly, we included solely 143 isolates presenting MDR phenotype from patients with wide range of ages, from birth to 88 years. The results obtained were consistent with those of the previous studies, according to which the mean age was varying between 56 and 62 years and ranged from birth to 91 years [26, 27, 30, 31, 33].

In the present study, the distribution of Enterobacterales resistant to 3GC and/or carbapenems according to the nature of the sample revealed that majority of the isolates were the causative agents for urinary tract infections. This corroborates the findings of other studies that reported multidrug-resistant Enterobacterales as being predominantly isolated from urine samples [24, 30, 31, 34, 35]. However, others have shown that the respiratory tract (54.2%) and blood (56.49%) are the main sources of MDR isolates [26, 27, 29].

Additionally, Enterobacterales resistant to 3GC and/or carbapenems exhibited a high resistance rate to the different classes of antibiotics tested, including trimethoprim/sulfamethoxazole, norfloxacin, gentamicin, and piperacillin-tazobactam. These results are consistent with those of the other multidrug resistance studies in Morocco and internationally [26, 35, 36]. However, when comparing our results to those of our previous studies [37], it is noteworthy that our susceptibility tests to carbapenems among MDR isolates showed an increase in resistance rate from less than 1% for imipenem and 7.2% for ertapenem in 2010 to 23 and 40%, respectively. In the present study, the resistance to ertapenem has been multiplied thrice for Enterobacterales and by 20 times for *K. pneumoniae*. Meanwhile, the frequency of isolation of ESBL-producing Enterobacterales was 28.6% in 2010, which decreased to 16.3%, corresponding to the frequency found in the studies of Rabat [23, 37].

Further, the rate of CPE detected by molecular screening in our study was twice higher than the results reported in Iran (15%) [38, 39]. However, this rate is lower than the rate of carbapenemase producers (85.5%) reported in Morocco by Loqman et al. [26]. Therefore, the variation in the prevalence of CPE in other studies may be due to inclusion criteria and the patterns in the utilization of carbapenems.

For the MDR phenotype in our study, *K. pneumoniae* was also the prevalent carbapenemase-producer and this finding is consistent with that of the previous study [26, 40]. After the first report of carbapenem-resistant OXA-48-positive *K. pneumoniae* isolated in Morocco in 2009, it became the most frequent carbapenemases in Morocco thereafter, representing 76.9% and 63.6% in 2012 and 2015, respectively [41–43]. Our study showed that 83.3% of carbapenemase-producing strains contained OXA-48, which confirms the increased isolation rate of this enzyme in Rabat Morocco. In connection with the neighboring countries, blaOXA-48 is the most prevalent carbapenemase-encoding gene (89.6%), followed by blaNDM-1 gene (10.4%) in Algeria [44]. The spread of OXA-48-producing *K. pneumoniae* in the Mediterranean area had reached an endemic level in Turkey, Malta, Spain, Lebanon, Tunisia, Morocco, and Libya [45].

Moreover, the molecular screening results revealed that 16.7% of carbapenemase-producing strains contained blaNDM. In this study, all blaNDM genes were produced by *K. pneumoniae*, and the majority of blaNDM genes were blaNDM-1 type. The blaNDM-1 was reported in 2011 in Morocco from three isolates positive for blaNDM, and their DNA sequencing revealed that they produce NDM-1 [46]. However, carbapenem resistance was not initiated by a metallo-beta-lactamase enzyme in our study, where strains were 83.3% producers of OXA-48, while in Marrakech, strains were metallo-beta-lactamase producers (mainly NDM) and OXA-48-like producers in 43.82% and 41.75%, respectively [47].

One of the reasons for the high spread of OXA-48 compared to the spread of NDM can be attributed to a highly transmissible plasmid harboring OXA-48 gene, which is known as pOXA-48a. The transfer frequency of pOXA-48a is 50 times higher than those of the similar pNDM-OM IncL plasmids with blaNDM-1 gene [48].

For RepA replicase gene, further studies to analyze the other genes (repA, traU, and parA genes) are required before coming to any valid conclusion [48]. Future bacterial analyses with regard to resistome and virulome content, clonality, and plasmid support are prerequisite to explain these variations in the distribution of OXA-48- and NDM-producing Enterobacterales in the Moroccan hospitals. However, our results demonstrate an important diffusion of blaOXA-48-producing carbapenem-resistant*K. pneumoniae* and may serve as a guide in infection control measures. The major limitation in this study is the lack of sequencing of blaOXA-48- and blaNDM-producing strains.

## 5. Conclusion

The emergence of carbapenemase-producing Enterobacterales in hospitals and also in the community remains a real public health problem in Morocco. In the present study, the occurrence of carbapenem resistance was found to be high with numerous ESBL carriages, coupled with carbapenemase production by *K. pneumoniae*, followed by *E. cloacae.* The major carbapenemase producers are CPE of OXA-48 and NDM-1 carbapenemase. The control of this phenomenon does not necessarily require the discovery of novel antibacterial agents, but rather the strict observance of simple hospital hygiene measures and optimal utilization of antibiotics. In addition, the early detection of carbapenemases is an important epidemiologic and therapeutic task in the clinical laboratory to prevent the spread of carbapenemase-producing strains and aid the adjustment of appropriate treatment options. Therefore, we recommend and encourage the implantation of carbapenemase detection platform in all hospital settings and private laboratories to estimate the true burden of CPE.

## Figures and Tables

**Table 1:** Sequences of primers used for multiplex PCR.

PCR name	Targeted gene	Primer name	Sequence (5′ to 3′ end)	Amplicon size (bp)	Melting temperature (°C)	Reference
Multiplex 1	*bla*_KPC_ type	Forward	TGTTGCTGAAGGAGTTGGGC	340	56	[15]
		Reverse	ACGACGGCATAGTCATTTGC			
	*bla*_OXA-48-like_ including OXA-199 and OXA-370	Forward	AACGGGCGAACCAAGCATTTT	597		[15]
		Reverse	TGAGCACTTCTTTTGTGATGGCT			
	RepA	Forward	GACATTGAGTCAGTAGAAGG	905		[16]
		Reverse	CGTGCAGTTCGTCTTTCGGC			
	KPC	Forward	CTGTCTTGTCTCTCATGGCC	796		[17]
		Reverse	CCTCGCTGTGCTTGTCATCC			

Multiplex 2	*bla*_VIM_ type	Forward	CGCGGAGATTGARAAGCAAA	247	56	[15]
		Reverse	CGCAGCACCRGGATAGAARA			
	VIM2	Forward	ATGTTCAAACTTTTGAGTAAG	801		[18]
		Reverse	CTACTCAACGACTGAGCG			
	NDM-1	Forward	CATTTGCGGGGTTTTTAATG	998		[19]
		Reverse	CTGGGTCGAGGTCAGGATAG			

Multiplex 3	*bla*_NDM-1_	Forward	TAAAATACCTTGAGCGGGC	439	52	[15]
		Reverse	AAATGGAAACTGGCGACC			
	*bla*_IMP_ variants except, IMP-3, IMP-16, IMP-27, IMP-31, IMP-34, and IMP-35	Forward	GAGTGGCTTAATTCTCRATC	183		[20]
		Reverse	CCAAACYACTASGTTATCT			
	*bla*OXA-23-like	Forward	GTGGTTGCTTCTCTTTTTCT	736		[15]
		Reverse	ATTTCTGACCGCATTTCCAT			

Multiplex 4	OXA-58-like	Forward	TGGCACGCATTTAGACCG	507	52	[21]
		Reverse	AAACCCACATACCAACCC			
	VIM1	Forward	AGTGGTGAGTATCCGACAG	261		[20]
		Reverse	ATGAAAGTGCGTGGAGAC			
	NDM	Forward	GGTTTGGCGATCTGGTTTTC	621		[21]
		Reverse	CGGAATGGCTCATCACGATC			

Multiplex 5	OXA-23-like	Forward	GATCGGATTGGAGAACCAGA	501	52	[21]
		Reverse	ATTTCTGACCGCATTTCCAT			
	OXA-24-like	Forward	TTCCCCTAACATGAATTTGT	1024		[21]
		Reverse	GTACTAATCAAAGTTGTGAA			
	OXA-51-like	Forward	TAATGCTTTGATCGGCCTTG	353		[21]
		Reverse	TGGATTGCACTTCATCTTGG			

For degenerate primers: R = A or G; S = G or C; Y = C or T.

**Table 2:** Prevalence of carbapenemase-encoding genes.

Bacteria isolates	RepA	*bla*_OXA-48-_like	NDM-1	NDM	*bla*_NDM-1_
*K. pneumoniae*	26	29	5	7	7
*E. cloacae*	6	5	—	—	—
*E. coli*	—	1	—	—	—
Total of positive isolates	32	35	5	7	7

**Table 3:** Matrix correlation between PCR and the immune-chromatographic assays.

	Number of positive results on flow lateral test	Number of negative results on flow lateral test
Number of isolates positive by molecular screening	OXA-48-CPE (*n* = 34)	OXA-48-CPE (*n* = 1)
	NDM-CPE (*n* = 6)	NDM-CPE (*n* = 1)

Number of isolates negative by molecular screening	OXA-48-CPE (*n* = 0)	OXA-48-CPE (*n* = 101)
	NDM-CPE (*n* = 0)	NDM-CPE (*n* = 101)

*CPE stands for carbapenemases-producing Enterobacterales. N is the number of CPE-positive harboring OXA-48 or NDM genes.*

## Data Availability

The data for the current study are available from the corresponding author on reasonable request.
